# StripeDiff: Model-based algorithm for differential analysis of chromatin stripe

**DOI:** 10.1126/sciadv.abk2246

**Published:** 2022-12-07

**Authors:** Krishan Gupta, Guangyu Wang, Shuo Zhang, Xinlei Gao, Rongbin Zheng, Yanchun Zhang, Qingshu Meng, Lili Zhang, Qi Cao, Kaifu Chen

**Affiliations:** ^1^ Department of Cardiology, Boston Children’s Hospital, Boston, MA 02115, USA.; ^2^ Department of Pediatrics, Harvard Medical School, Boston, MA 02115, USA.; ^3^ Houston Methodist Hospital Research Institute, Houston, TX 77030, USA.; ^4^ Department of Urology, Feinberg School of Medicine, Northwestern University, Chicago, IL 60611, USA.; ^5^ Robert H. Lurie Comprehensive Cancer Center, Northwestern University Feinberg School of Medicine, Chicago, IL 60611, USA.; ^6^ Broad Institute of MIT and Harvard, Boston, MA 02115, USA.; ^7^ Dana-Farber/Harvard Cancer Center, Boston, MA 02115, USA.

## Abstract

Multiple recent studies revealed stripes as an architectural feature of three-dimensional chromatin and found stripes connected to epigenetic regulation of transcription. Whereas a couple of tools are available to define stripes in a single sample, there is yet no reported method to quantitatively measure the dynamic change of each stripe between samples. Here, we developed StripeDiff, a bioinformatics tool that delivers a set of statistical methods to detect differential stripes between samples. StripeDiff showed optimal performance in both simulation data analysis and real Hi-C data analysis. Applying StripeDiff to 12 sets of Hi-C data revealed new insights into the connection between change of chromatin stripe and change of chromatin modification, transcriptional regulation, and cell differentiation. StripeDiff will be a robust tool for the community to facilitate understanding of stripes and their function in numerous biological models.

## Teaser

A statistical framework accurately detects differential chromatin stripes that regulate gene expression changes between samples.

## INTRODUCTION

The eukaryotic DNA is folded into hierarchical chromatin domains at multiple genomic scales in the three-dimensional (3D) space. The development of chromosome conformation capture (3C) assay (*1*) and its variants, including 4C (*2*, *3*), 5C (*4*), Hi-C (*5*), ChIA-PET (*6*), single-cell Hi-C (*7*), and many others, has enabled measuring 3D chromatin contact frequency at a high resolution. At a large scale, individual chromatins occupy different nuclear territories (*8*), an observation that has a long history and is consistent with recent 3D genomic data (*5*). At a finer scale, the 3D architecture of a chromatin falls into different compartments, e.g., the active compartment A and repressive compartment B (*5*), or a few subcompartments associated with biological functions such as the timing of DNA replication (*9*). Genomic regions that belong to the same compartment type tend to have higher contact frequency with each other, leading to a checkerboard-like pattern in a 2D map of contact frequency across the genome. Facilitated by increased resolutions of genomic methods to investigate chromatin 3D architecture, chromatin was further found to fold into topologically associated domains (TADs) (*10*, *11*), in which the contact between DNA from the same TAD is more frequent than between DNA from different TADs. TAD differs from the larger-scale A and B compartments in that they do not necessarily form an alternating checkerboard-like pattern in the 2D map of contact frequency, and several TADs often reside within a single contiguous compartment. In about 50% of the TADs, contact frequency was found to be higher between the two boundaries of a TAD than between loci within a TAD, suggesting a more frequent existence of chromatin loop between the boundaries (*9*). Recent studies have identified the chromatin stripe (*12*), which appears as higher contact frequency between one boundary and many loci within a TAD than between other pairs of loci in the TAD. Meanwhile, there are also some cases in which a strong loop or stripe can appear without a TAD being clearly observed. In addition to loop and stripe, frequently interacting region (FIRE) was found to be associated with enhancers near tissue-specific genes (*13*). In contrast to loop and stripe that tend to be observed at the boundary of TAD, FIRE was found near the center of TAD.

Loop extrusion was proposed as a key mechanism of chromatin folding (*14*). Loop extrusion is closely related to some earlier concepts (*15*), e.g., progressive loop enlargement (*16*), loop expansion (*17*), facilitated tracking (*18*), and reeling (*19*). In loop extrusion, a ring-like structural maintenance of chromosomes (SMC) complex binds on a chromatin to extrude a chromatin loop from the inside of the ring. The extrusion continues until halted by proteins that bind on the chromatin at the loop boundaries (*14*). For example, the convergent binding sites of CCCTC-Binding Factor (CTCF) at the two boundaries of a chromatin loop or TAD were known as insulators that halt loop extrusion (*20*). SMC complexes may stochastically load or dissociate at sites within a TAD but is hard to pass the boundary; therefore, contact frequency can be uniformly high between sites within a TAD but becomes low between TADs when analyzed in a cell population; furthermore, a loop between TAD boundaries might be observed in the 2D contact map due to increased length of SMC processivity on the DNA to arrive at the boundaries before disassociation (*21*). The extrusion model is also supported by recent real-time imaging analysis, which indicated that SMC complexes such as condensin and cohesin extruded a DNA loop in an adenosine triphosphate (ATP)–dependent manner (*22*–*24*). Therefore, the extrusion model was found to be remarkably successful at explaining these important features of 3D chromatin folding (*25*), although the chromosome territories and compartments were proposed to arise from a different mechanism independent from SMC complexes (*26*).

In contrast to the compartment, TAD, and loop architectures that have been known for a while, the stripe architecture is a relatively new observation. The formation of stripe could also be well explained by the extrusion model, as it could be a result of extruding chromatin from one side of the SMC ring structure, while the other side was retained on a stable anchor (*12*). Consistently, recent in vitro single-molecule experiments have reported cases in which the yeast condensin (*22*), *Xenopus* SMC complex (*27*), and human SMC complex (*28*) extrude a chromatin loop in a single-sided manner. A stripe was found to connect a promoter to a long stretch of enhancers and deregulate immunoglobulin heavy locus (IGH)–translocated oncogene in plasmacytomas (*12*). It was reported that stripe formation is associated with the functional state of a differentiating cell and regulated by the binding of CTCF and cohesion proteins on lineage-specific enhancer elements (*29*).

Whereas a couple of methods were reported to define stripes in a single sample (*12*, *29*), there are few algorithms and statistical models, except for fold change analysis (*12*), to quantify the difference of a stripe between samples. In this study, we developed the bioinformatics toolkit StripeDiff to systemically detect differential stripes across the genome. StripeDiff represents a new statistical model to test the significance of difference in each stripe between samples. To evaluate the performance of StripeDiff, we developed a simulation method to generate mocked differential stripes for which the background truth is known. We further compiled a benchmark system to test StripeDiff based on real Hi-C data. The simulation data and benchmark data both demonstrated robust performance of StripeDiff. We next applied StripeDiff to 12 different Hi-C datasets and revealed insights into the biological implication of differential stripes.

## RESULTS

### Development of the StripeDiff algorithm to detect differential stripes between Hi-C samples

**Fig. 1.**
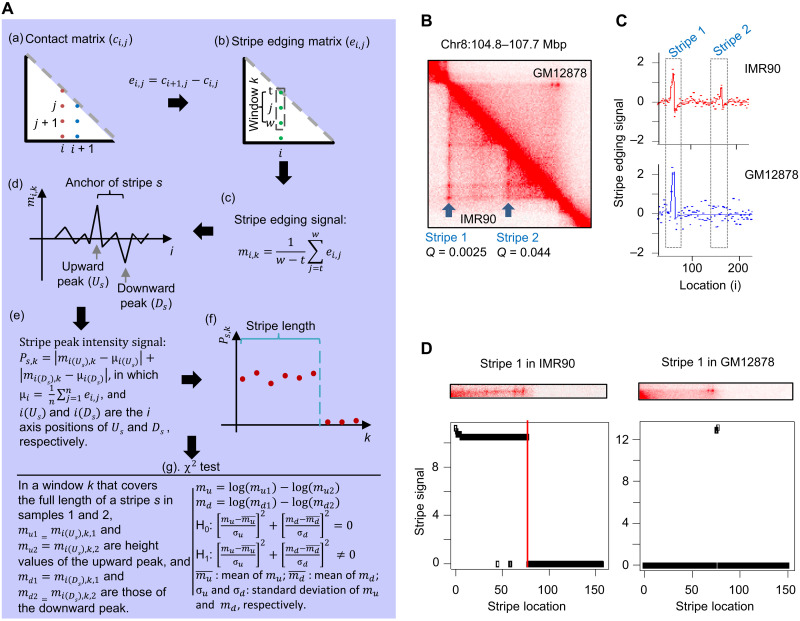
The StripeDiff algorithm to detect differential stripes. (**A**) Flowchart to show the workflow of StripeDiff. (**B**) Heatmap of chromatin contact frequency showing two stripes observed in IMR90 cells and weakened in GM12878 cells. (**C**) Stripe edging signal of the same region from (B) in IMR90 cells (top) and GM12878 cells (bottom). (**D**) Heatmaps of chromatin contact frequency (top) and stripe peak intensity signal (bottom) to show examples of stripe length estimation.

The first function we developed in StripeDiff is to define the anchor sites of individual stripes across the genome in each Hi-C contact map. The input data for StripeDiff are normalized Hi-C contact maps. We define *c*_*i*, *j*_ as the interaction frequency between a pair of genomic loci *i* on the *x* axis and *j* on the *y* axis (Fig. 1A, step a). We can then denote the whole contact map as *C* = {*c*_*i*, *j*_}, in which the *i* and *j* each could vary between 1 and the genome size *N*. Therefore, the contact map is a symmetric square, and we will only need to analyze one of the two triangles divided by the diagonal between sites *c*_1, *N*_ and *c*_*N*,1_. Considering a vertical stripe in the bottom left triangle, our algorithm will first define the left and right edges of the stripe (define the vertical domain of the stripe). A key feature of a stripe’s edge is a big difference in the value of *c*_*i*, *j*_ between the left and right sides of the edge, e.g., for the left edge, the values at the right side (located within the stripe) will be larger than the values at the left side (located outside of the stripe). Therefore, we defined a stripe edging signal matrix *E* = {*e*_*i*, *j*_}, in which *e*_*i*, *j*_ = *c*_*i* + 1, *j*_ − *c*_*i*, *j*_ (Fig. 1A, step b). Assuming that the top and bottom edges of this vertical stripe are located at *j* = *t* and *j* = *w*, respectively, we then only need to analyze the values in the vertical window *k* located between *j* = *t* and *j* = w (Fig. 1A, step b). We next defined a vector *M_k_* = {*m*_*i*, *k*_}, in which *m*_*i*, *k*_ is the average of all *e*_*i*, *j*_ values from *j* = *t* to *j* = *w*; therefore, $m_{i,k}=\frac{1}{w-t}\sum_{\;j=t}^{w}e_{i,j}$ (Fig. 1A, step c). We then plot the values *m*_*i*, *k*_ as a function of the positions *i* (Fig. 1A, step d). Intuitively, *m*_*i*, *k*_ will be a positive value when the position *i* is on the left edge of a vertical stripe, whereas it will be a negative value when the position *i* is on the right edge of a vertical stripe and be a value close to zero when the position *i* is inside or outside of the stripe. Therefore, an upward peak *U_s_* and a downward peak *D_s_* will be observed at the left and right edges of a stripe *s*. We tested this idea on two stripes observed in the fibroblast cell IMR90 by visual inspection (Fig. 1B). As expected, the upward and downward peaks were observed at the edges of each stripe in IMR90 (Fig. 1C). Last, the anchor site of the stripe will be defined as the region from the left edge indicated by the upward peak *U_s_* to the right edge indicated by the downward peak *D_s_* (Fig. 1A, step d).

The next function we developed in StripeDiff is to estimate the length of each stripe (define the horizontal domain of the stripe). We first vertically slid the start point of the window *k* described above downward from the diagonal to the bottom of the stripe edging matrix (Fig. 1A, step b). We further defined a peak intensity score *P*_*s*, *k*_ for a stripe *s* in each window *k* by normalizing and aggregating the edging signal *m*_*i*, *k*_ at the upward peak *U_s_* and downward peak *D_s_* (Fig. 1A, step e; see Materials and Methods). We then plot the score *P*_*s*, *k*_ as a function of *k* and used the change point detection (CPD) method (*30*) to divide the peak intensity scores into the high versus low peak intensity segments (Fig. 1A, step f). This result allows us to define aggregated length of high peak intensity segments as the stripe length (Fig. 1D).

After the detection of individual stripes in each single Hi-C sample, the third function in StripeDiff defines differential stripes between a pair of samples (Fig. 1A, step g). For a stripe, we evaluate the change of stripe edging signal between a pair of samples 1 and 2 at both the upward and the downward peaks by a chi-square test. Given stripe edging signals *m*_*u*1_ versus *m*_*u*2_ in the two samples at the upward peak and *m*_*d*1_ versus *m*_*d*2_ at the downward peak, the null hypothesis is that *m*_*u*1_ = *m*_*u*2_ and *m*_*d*1_ = *m*_*d*2_. The normalized values in a Hi-C contact matrix can be modeled as normally distributed Gaussian random variables (*31*, *32*). To have the distribution closer to a normal distribution (fig. S1), we further transformed the values to the log scale (Fig. 1A, step g). To evaluate the significance of difference in the stripe edging signal, we performed chi-square test to determine the *P* value (different from the popular application of chi-square test on a 2 × 2 contingency table; see Materials and Methods) and further adjusted the *P* values based on the Benjamin-Hochberg method.

### Simulation analysis demonstrated robust performance of StripeDiff

**Fig. 2.**
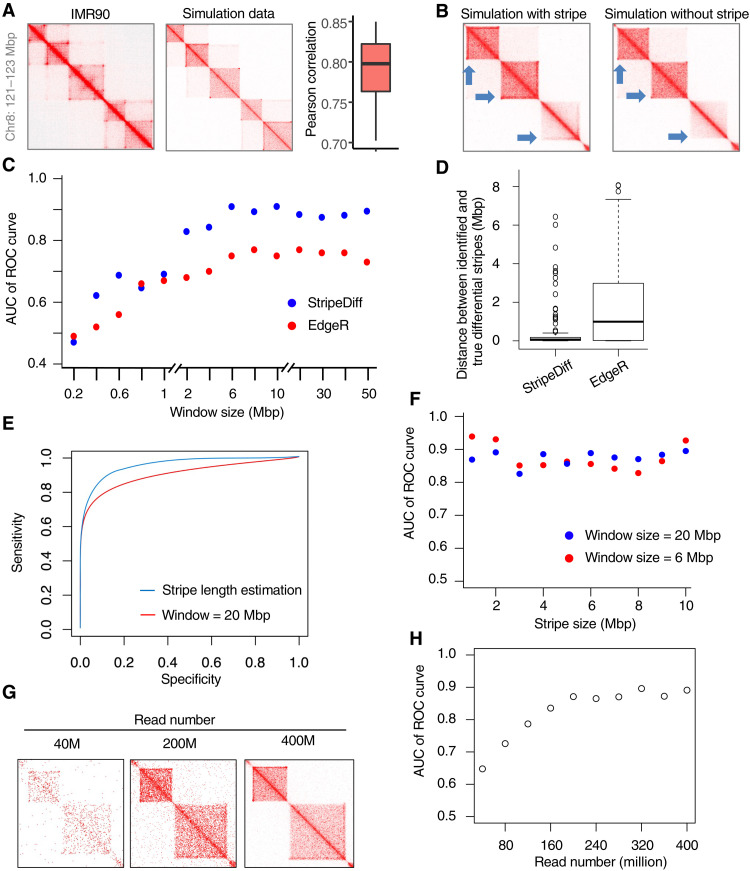
Simulation data to show the robust performance of StripeDiff. (**A**) Heatmaps to show chromatin contact frequency in real Hi-C data from IMR90 cells (left) and in simulated data (middle), and boxplot to show correlation of chromatin contact frequency between simulated and real data (right). (**B**) Heatmaps showing simulated chromatin contact frequency with (left) and without stripes (right). Blue arrows indicate stripe loci. (**C**) AUC values of ROC plotted as a function of window sizes used by individual algorithms to detect differential stripes. (**D**) Boxplot to show distance between true differential stripes and differential stripes identified by individual algorithms. (**E**) ROC showing the performance of StripeDiff algorithm in defining differential stripes based on a fixed 20-Mbp window or the detected stripe area. (**F**) AUC values of ROC plotted as a function of mean length of analyzed stripes to show performance of StripeDiff in detecting differential stripes. (**G**) Heatmaps of chromatin contact frequency in simulated Hi-C data with different sequencing depths. (**H**) AUC values of ROC plotted as a function of sequencing depth in simulated Hi-C data to show performance of StripeDiff in detecting differential stripes.

To assess the performance of StripeDiff, we developed SimuStripe, a pipeline to generate simulation data in which stripes were embedded (see Materials and Methods) (fig. S2). Because stripes often co-occur with loops and TADs, SimuStripe further embeds loops and TADs along with stripes. For the simulation data to mimic real Hi-C data, we used real Hi-C data as a template for generation of simulation data so that the distribution of simulated location, size, and average contact frequency in individual TADs, loops, and stripes will be close to those observed in real data. Manual inspection of individual genomic regions in the simulated data confirmed the consistency of stripes, loops, and TADs between the simulated and real data (Fig. 2A, left and middle). Correlation analysis also indicated great similarity between the simulated and real data (Fig. 2A, right). Therefore, SimuStripe simulated chromatin contact matrices with high fidelity.

Next, we applied StripeDiff to a pair of simulated Hi-C samples, in which we knew the background truth of differential stripes (Fig. 2B). We first analyzed the effect of the size of the window *k* on the performance of StripeDiff in recapturing the anchor locations of simulated differential stripes. For each tested window size, we repeated the simulation 1000 times, with each time simulated a random genomic region, for which the region size is equal to the window size. Two replicates were simulated for each region in each of two simulated samples. This resulted in four simulated datasets for two samples. We then detected differential stripes between the two samples. We plotted receiver operating characteristic (ROC) curve and calculated the area under the curve (AUC) of ROC curve to evaluate the performance. For comparison, we also customized the edgeR algorithm for differential stripe analysis (see Materials and Methods) and compared it to our StripeDiff algorithm. EdgeR was developed for differential gene expression analysis (*33*) but was also used to perform differential analysis of chromatin contact frequency in TADs or loops (*31*, *34*). The results showed that the AUC of the ROC curve of StripeDiff increased from 0.48 to 0.9 when the window size increased from 0.2 to 6 million base pairs (Mbp) and thereafter appeared as being saturated, while AUC of the ROC curve of edgeR ranges from 0.49 to 0.81 (Fig. 2C). To further test the reliability of StripeDiff, we calculated the distance between true differential stripes and the identified differential stripes. We found that the differential stripes identified by StripeDiff were located near the true differential stripes, while the differential stripes identified using edgeR are significantly (Wilcoxon test *P* < 1 × 10^−16^) more distant from the true differential stripes (Fig. 2D). To evaluate the effect of data noise on the performance of StripeDiff, we used published algorithms (*35*), which added two types of noise to the simulation data. The two noise types included noise associated with genomic distance effect and noise associated with random ligations generated by the Hi-C protocol. The AUC of the ROC curve decreased slightly when we increased the percentage of noise reads from 0 to 50% (noise-to-signal ratio from 0 to 1). However, StripeDiff performed better than EdgeR at all these noise ratios (fig. S3). The default algorithm in StripeDiff defines the length of each stripe, thus detecting differential stripes based on the full-length region of each stripe rather than analyzing a window area with the same window size for all stripes. StripeDiff showed a slightly better performance by analyzing the full-length region of each stripe (Fig. 2E), although analyzing a window area with a fixed window size at all stripes can be faster (fig. S4).

We further tested whether the performance of StripeDiff will be different for stripes with different lengths. We used SimuStripe to generate differential stripes, of which the length ranged from 1 to 10 Mbp. We then identified differential stripes using 6- and 20-Mbp window sizes. The results showed that StripeDiff has a similarly robust performance on stripes with different lengths, and there is little difference in performance when different window sizes were used in the algorithm (Fig. 2F). However, for very short stripes, it is slightly better to use a smaller window size (fig. S5, A and B). We also designed an alternative algorithm to calculate edging signal as log fold change rather than the subtraction value. The performance of this alternative algorithm based on log fold change is still good, although the default algorithm based on subtraction appeared to be better (fig. S5, C and D). Moreover, we investigated whether StripeDiff is sensitive to sequencing depth in Hi-C experiments. We downsampled the sequencing depth of simulation data from 400 million to 40 million intrachromosomal reads (Fig. 2G). The results showed that the AUC of the ROC curve increased from 0.65 to 0.89 when the sequencing depth increased from 40 to 200 Mbp and became saturated thereafter (Fig. 2H). The effect of sequencing depth did not change much with the length of the analyzed stripes (fig. S6, A and B). Last, although we designed StripeDiff with a focus on differential analysis of stripes between samples, it also showed better performance in detection of stripes in a single sample when compared to the reported algorithms Zebra (*12*) and StripeNN (*36*) (fig. S7). These results indicated that StripeDiff is a robust algorithm for the detection of differential stripes between simulated Hi-C samples.

### StripeDiff showed a reliable performance on real Hi-C data

**Fig. 3.**
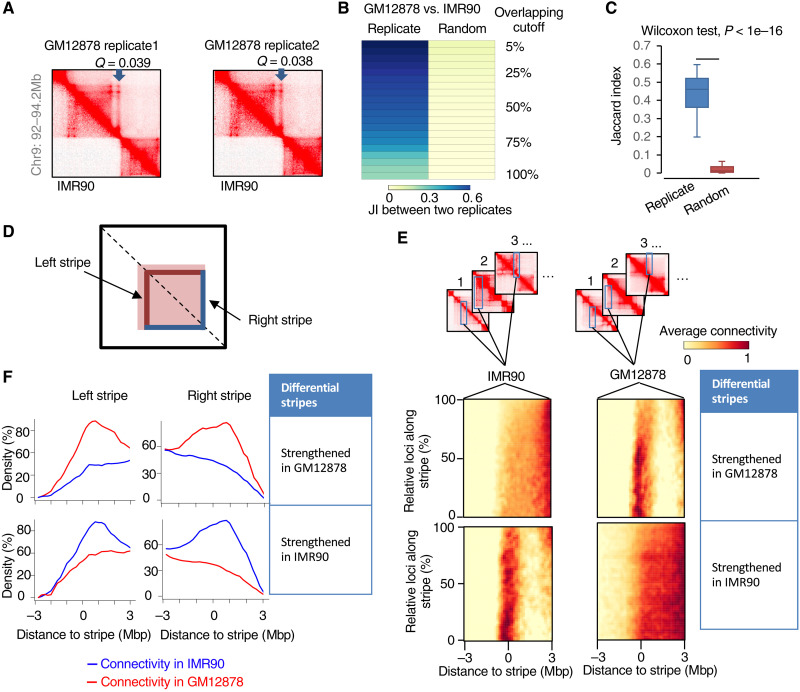
StripeDiff showed reliable performance on real Hi-C data. (**A**) Heatmaps of chromatin contact frequency to show differential stripes between IMR90 and two replicates of GM12878 cells. (**B** and **C**) Heatmap (B) and boxplot (C) to show Jaccard index values of differential stripes between replicates. Jaccard index values are calculated by different overlapping cutoffs. (**D**) Cartoon showing the definition of left and right stripes. (**E**) Heatmaps showing the average contact frequencies of differential stripes strengthened in GM12878 or IMR90. (**F**) Average contact frequencies for left and right differential stripes strengthened in GM12878 or IMR90.

To evaluate the reproducibility of differential stripe identification in real Hi-C data, we compared IMR90 to two replicates of GM12878 at a resolution of 10 kilo–base pair (kbp). We manually checked individual differential stripes identified by StripeDiff and observed that many differential stripes were each identified in both replicates (Fig. 3A). We denoted the differential stripes between GM12878 replicate 1 and IMR90 as the differential stripe set 1 and denoted the differential stripes between GM12878 replicate 2 and IMR90 as the differential stripe set 2. We used the Jaccard index, a similarity coefficient defined as the size of the intersection between differential stripe set 1 and set 2 divided by the size of their union, to measure the reproducibility of the identified differential stripes between the two replicates. We tried different cutoffs to define intersection between two stripes from the two groups and consistently found that the Jaccard index scores between the two replicates are significantly larger than expected by random chance (*P* < 1 × 10^−16^, Wilcoxon test; Fig. 3, B and C). As a Hi-C contact map is symmetric, we defined the vertical stripes in the left bottom triangle of contact maps as the left stripes and the vertical stripes in the right upper triangle (i.e., horizontal stripes in the left bottom triangle) of contact maps as the right stripes (Fig. 3D). We calculated the average frequency of chromatin contact in the region from 3 Mbp upstream to 3 Mbp downstream of the anchor sites for individual differential stripes. The results showed that, for both left and right stripes, the density of chromatin interaction in strengthened stripes is significantly higher than the density in weakened stripes, as indicated by both pileup heatmaps (Fig. 3E) and curve plots (Fig. 3F). These results demonstrated reliable performance of StripeDiff on real Hi-C data.

### Differential stripes detected by StripeDiff were associated with changes of CTCF binding on chromatin

**Fig. 4.**
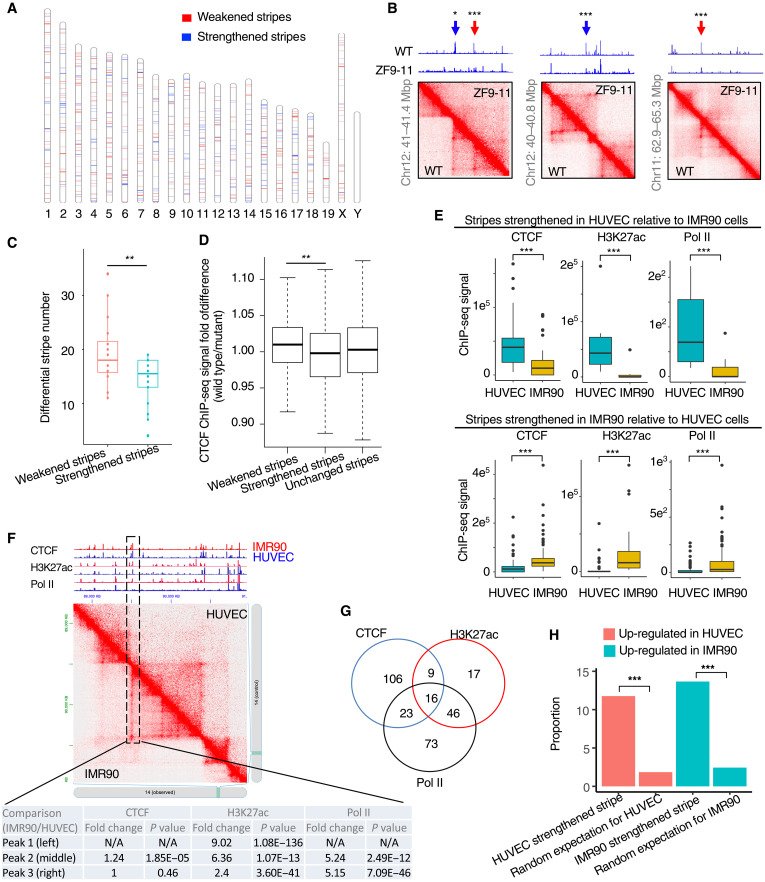
Differential stripes are linked to changes of chromatin state and gene expression. (**A**) Chromosome map showing genomic locations of differential stripes induced by CTCF ZF9-11 mutation in the cell line CH12. (**B**) Heatmaps of chromatin contact frequency (bottom) and Genome Browser tracks (top) of ChIP-seq signal for CTCF in CH12 cells. Blue arrows indicate locations of differential stripes reported in the original publication of the data. Red arrows indicate differential stripes newly detected by StripeDiff. Genome Browser tracks were based on bigwig files downloaded from the original publication of the data. (**C**) Boxplot to show numbers of differential stripes induced by CTCF ZF9-11 mutation on individual chromosomes in CH12 cells. (**D**) Boxplot to show fold change of CTCF binding signal at anchors of individual groups of stripes in response to CTCF ZF9-11 mutation in CH12 cells. (**E**) Boxplots showing ChIP-seq signal of individual chromatin components in anchors of differential stripes detected between HUVEC and IMR90 cells. (**F**) Heatmap of chromatin contact frequency (bottom) and Genome Browser tracks (top) of ChIP-seq signal. (**G**) Venn diagram to show number of differential stripes positively associated with the changes of CTCF, H3K27ac, or Pol II ChIP-seq signals between HUVEC and IMR90 cells. (**H**) Bar plot to show the percentage of differential genes on differential stripes defined between HUVEC and IMR90 cells. The value for random expectation is calculated as 100 × *N_r_*/*N_e_* and *N_r_* = *N_s_* × *N_e_*/*N*, where *N* is the number of all genes in the genome, *N_s_* is the number of all genes in differential stripes, and *N_e_* shows differential expression. *P* value is calculated by Poisson test (B and F), Wilcoxon test (C to E), and Fisher’s exact test (H). *P < 0.05, **P < 0.01, and ***P < 0.001.

Previous studies suggested that stripes are formed because an SMC complex extrudes a chromatin loop by sliding only one of the two loop ends (*12*, *37*), with the other end being the stripe anchor. It is reported that the binding of CTCF is enriched on the stripe anchor to halt the sliding of chromatin at the associated loop end (*12*, *29*). Therefore, we investigated whether the reduction of CTCF binding could lead to the weakening of stripes. We downloaded a pair of Hi-C datasets generated for CH12 cells with weakened CTCF binding affinity due to a mutation in CTCF zinc fingers (ZFs) and for wild-type CH12 cells. In total, StripeDiff detected 652 differential stripes between the wild-type and CTCF mutant CH12 cells. These differential stripes are distributed on all chromosomes of the mouse genome (Fig. 4A). Visual inspection confirmed that StripeDiff successfully recaptured differential stripes reported as examples previously (blue arrows in Fig. 4B) (*12*), and further detected new differential stripes in the same set of data (red arrows in Fig. 4B). Consistent with the knowledge that the binding of CTCF is associated with the formation of stripes, the number of weakened stripes in the mutant cells relative to the wild types is significantly larger than the number of strengthened stripes on individual chromosomes (*P* = 0.0083, Wilcoxon test) (Fig. 4C). By manually inspecting the CTCF chromatin immunoprecipitation sequencing (ChIP-seq) signals at differential stripes, we observed that the weakened stripes tend to show reduced binding of CTCF, as indicated by weakened ChIP-seq signals (Fig. 4B). This observation is consistent with results from a statistical analysis of all weakened or strengthened stripes. Compared to the stripes that are weaker in wild-type cells relative to the CTCF mutant cells, the stripes that are stronger in wild-type cells are more likely to show stronger CTCF ChIP-seq signals in the wild-type cells relative to the mutant cells (Fig. 4D). In addition, the number of oriented motifs is greater than the number of opposite motifs at the anchor locations of differential stripes (fig. S8). Together, these results from differential stripes defined by StripeDiff confirmed an important role of CTCF in stripe formation.

### Differential stripes detected by StripeDiff were associated with changes of chromatin state

It was reported that the formation of stripe is associated with enhancer activity and RNA polymerase II (Pol II) elongation (*12*, *29*). Thus, we further analyzed how the ChIP-seq signals of Pol II and the enhancer marker H3K27ac change at differential stripes. By applying StripeDiff to Hi-C data from IMR90 and endothelial cell HUVEC (human umbilical vein endothelial cell), we detected 718 differential stripes between these two cell types. Next, we analyzed ChIP-seq data for CTCF, H3K27ac, and Pol II from the two cell types at these differential stripes. The results are consistent with the knowledge that stripe strengthening is associated with increased ChIP-seq signals of CTCF, H3K27ac, and Pol II, whereas weakened stripes displayed a reduction of these signals (Fig. 4E).

By manually inspecting ChIP-seq signals at individual differential stripes, we observed that CTCF, H3K27ac, and Pol II did not always change at the same differential stripe. For instance, a stripe weakened in HUVEC relative to IMR90 could show a reduction of Pol II binding but little change of CTCF binding (Fig. 4F). We found 290 (40%) of the differential stripes positively associated with a change of CTCF, H3K27ac, or Pol II ChIP-seq signal, whereas only 16 (2%) of the differential stripes were positively associated with change of all these signals (Fig. 4G). Of all differential stripes, 154 (21%) showed a positive association with differential binding of CTCF, 88 (12%) showed a positive association with change of H3K27ac signal, and 158 (22%) showed a positive association with change of Pol II ChIP-seq signal (Fig. 4F). Together, these results indicated that there could be different mechanisms to cause strengthening or weakening of a stripe.

### Differential stripes detected by StripeDiff were associated with gene expression changes

As CTCF binding, enhancer activation, and RNA Pol II activity are all associated with transcriptional regulation of gene expression, we further investigated whether the differential stripes defined by StripeDiff are accompanied by changes of gene expression. We identified 7588 differentially expressed genes between HUVEC and IMR90 based on RNA-sequencing (RNA-seq) data from these two cell types. Our analysis indicated that 11.7% of the genes up-regulated in HUVEC relative to IMR90 overlapped with genes in stripes strengthened in HUVEC relative to IMR90, whereas only 1.8% overlap would be expected by chance (Fig. 4H). Similarly, 13.6% of the genes up-regulated in IMR90 relative to HUVEC overlapped with genes in stripes strengthened in IMR90 relative to HUVEC, whereas only 2.4% overlap would be expected by chance. The enrichment of up-regulated genes in strengthened stripes was still observed when the Hi-C data were normalized with different methods, although the degree of enrichment varied when different normalization methods were used (fig. S9A). The enrichment was also observed when we used different *P* value cutoffs to define differential genes (fig. S9B). These results indicated that the stripe formation is positively associated with activation of transcription.

### A landscape analysis using StripeDiff revealed association between differential stripes and cell lineage specification

**Fig. 5.**
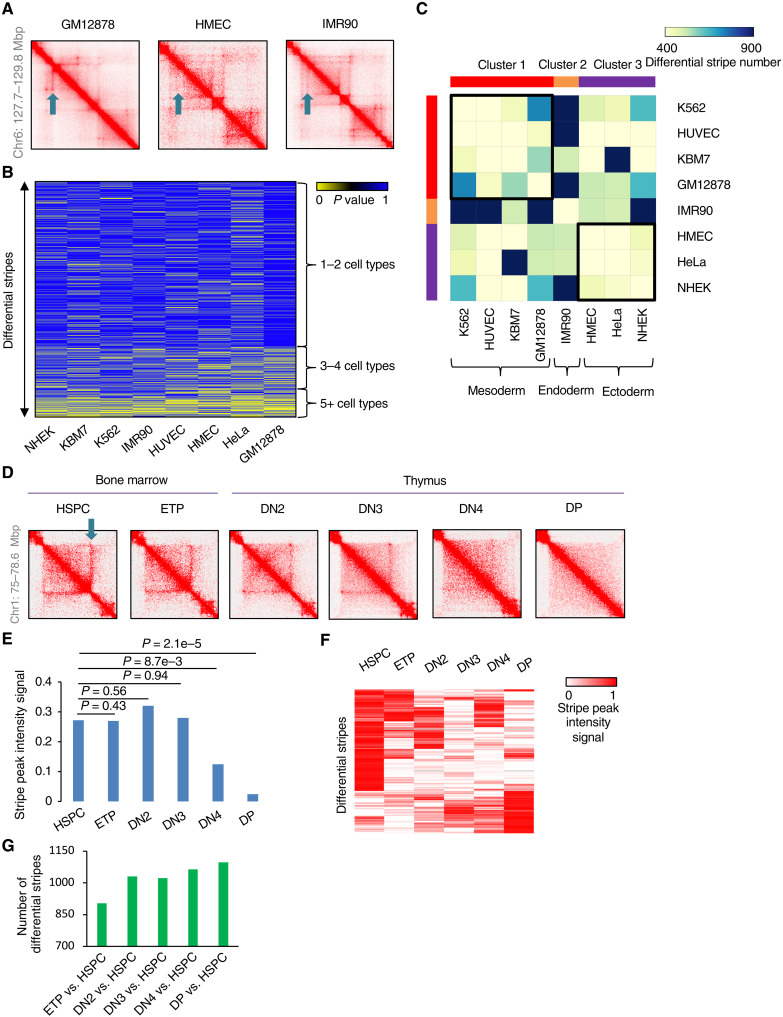
A landscape analysis indicated strong cell type specificity of differential stripes. (**A**) Heatmaps showing chromatin contact frequency at the same locus in GM12878, HMEC, and IMR90 cells. Arrows indicate stripe location. (**B**) Heatmap showing differential stripes from eight cell types. Each stripe in the heatmap was defined as differential in at least one pair of cell types from the eight cell types. (**C**) Heatmap showing numbers of differential stripes between each pair of cell types. (**D**) Heatmaps showing chromatin contact frequency within the same locus at six stages of T cell differentiation. Arrow indicates stripe location. (**E**) The bar plot shows the stripe peak intensity signals of the differential stripe observed in (D). The stripe peak intensity signal is *P_s,k_* as indicated in Fig. 1A. (**F**) Heatmap showing stripe signals of individual differential stripes at six stages of T cell differentiation. (**G**) Bar plot showing numbers of differential stripes between each of the other five stages and HSPC during T cell differentiation.

A recent investigation in embryonic stem cells and neural stem cells showed change of stripe during cell lineage specification (*29*). Little is known yet about the specificity of stripe strengthening or weakening among different cell types. One possibility is that stripes are highly conserved between cell types but might be specifically lost in a cell type; alternatively, it is possible that many stripes each appear in a specific cell type and are absent in most cell types. To test these hypotheses, we used StripeDiff to investigate the changes of stripes among Hi-C datasets generated by the same laboratory for eight different cell types (*9*), including IMR90, NHEK, HUVEC, HMEC, HeLa, GM12878, KBM7, and K562. By manual inspection of individual genomic regions, it was clear that some stripes could be cell type specific (Fig. 5A). We performed comparisons between each pair of these cell lines and divided the resultant 8003 differential stripes into three groups, i.e., stripes specifically strengthened in one or two cell types, three or four cell types, and at least five of the eight cell types (Fig. 5B). The results showed that 5693 (71%) of the differential stripes were strengthened in only one or two cell types, whereas only 637 (8%) were strengthened in five or more of these cell types. We next clustered the eight cell types based on the number of differential stripes between each pair. The cell types that have a closer developmental relationship appear to have fewer differential stripes and thus tend to cluster together (Fig. 5C), i.e., mesoderm cells K562, HUVEC, KBM7, and GM12878 were in the same cluster, whereas ectoderm cells HMEC, HeLa, and NHEK were clustered together, with the endoderm cell IMR90 clustered alone. These results suggested that most of the differential stripes are highly cell type specific and associated with cell lineage specification.

To further understand the dynamic change of stripes during the cell lineage specification, we analyzed Hi-C data generated at six different stages of T cell differentiation from hematopoietic stem and progenitor cell (HSPC) to CD4 and CD8 double-positive (DP) T cell (*38*). A visual inspection showed that the stripes could change gradually during T cell lineage specification (Fig. 5, D and E). We applied StripeDiff to compare between HSPC and DP cells and identified 1098 differential stripes of 5120 stripes (Fig. 5F). The number of differential stripes increased progressively during T cell lineage commitment (Fig. 5G). Together, these results further demonstrated a positive association between the changes of chromatin stripes and alternation of cell identities.

## DISCUSSION

The chromosomes in the nucleus are hierarchically folded into multiple layers of 3D architectures, such as chromosome territories, compartments, TADs, loops, and stripes (*39*–*43*). At each of these layers, individual units of the associated architecture are not randomly located across the genome (*44*). It was reported that a global flapping of A and B compartments played an important role in T cell lineage specification and in the process of cardiogenesis (*38*, *45*). Most TAD boundaries tend to remain stable across cell types, whereas a small subset of TAD boundaries could be cell type specific (*10*, *44*, *46*). Disruption of TAD boundary was reported to rewire gene-enhancer interactions and lead to distinct human limb malformations (*47*). Recent studies suggest that the stripe, a relatively new type of chromosome 3D architecture, is associated with both poised and active chromatin states (*12*, *29*). It is also reported that a subset of stripes was each strengthened or weakened after neuronal differentiation in comparison to embryonic stem cells (*29*). Whereas many bioinformatics tools have been developed to analyze the other architectures, no reported tool is available to statistically define differential stripes across a genome between samples.

In this study, we presented a set of computational tools, StripeDiff, to detect differential stripes between Hi-C samples. StripeDiff transforms the matrix of chromatin contact frequencies into a matrix of stripe edging signals, which enables us to identify the left and right edges of each stripe. It then used chi-square test to estimate the significance of the change of each stripe between two Hi-C samples. To evaluate the performance of StripeDiff, we performed simulation analyses that indicated an optimal performance of StripeDiff with its default parameters. In addition to the simulation experiments, we tested StripeDiff on real Hi-C data from the cell lines IMR90 and GM12878. The results showed that StripeDiff is highly reliable when applied to Hi-C data. We further used StripeDiff to analyze multiple public datasets and showed that the differential stripes defined by StripeDiff were associated with changes of CTCF binding, enhancer activation, RNA Pol II activity on chromatin, and gene expression. A landscape analysis of differential stripes among eight cell types indicated that cell type–specific stripe formation is more frequent than cell type–specific stripe loss.

Because there have been multiple mature tools to normalize Hi-C data, e.g., the Juicer (*48*), we did not further develop new normalization algorithm in StripeDiff. Instead, we suggest users to normalize the data using established methods and tools. The vanilla coverage normalization method (*5*) in the Juicer software is the default method in this article to normalize the input data to StripeDiff. We performed a comparison among the Knight-RuiZ (*49*), Sequential Component (*50*), vanilla coverage (*5*), and Vanilla-Coverage SQRT (*8*) methods for Hi-C data normalization based on the enrichment of up-regulated genes in strengthened stripes (fig. S9A). The results indicated that all these methods performed well and none of these methods always resulted in less enrichment of up-regulated genes in strengthened stripes. It might be intriguing to further investigate comprehensively in the future how the performance of StripeDiff might vary when different normalization methods are used. A critical challenge would be to develop a robust benchmarking system that provides a comprehensive set of golden standards to evaluate the performance on real Hi-C data.

The edging signal in StripeDiff is calculated as based on the local difference of contact frequency in the vertical or horizontal directions. This edging matrix is conceptually similar in spirit to the local horizontal and vertical approach adopted by Vian *et al.* (*12*). A key innovation in StripeDiff is that the edging matrix enabled identification of the two edges of a stripe so that the algorithm does not assume stripes to be the same in width. Different from StripeDiff, the algorithm of Vian *et al.* (*12*) assumed that a stripe had a fixed width of three bins and identified a stripe by comparing the three bins to a group of four bins next to each of the two edges. Because StripeDiff evaluates difference in edging signal based on values transformed to a log scale before subtraction, this is close to the fold change analysis performed by Vian *et al.* (*12*). The key difference is that StripeDiff analyzes the change of edging signal at the two boundaries of the stripe rather than the change of contact frequency within the stripe.

The current StripeDiff algorithm performs comparison between two conditions, with one sample from each condition. In the case of multiple replicates per condition, the aggregated Hi-C map of each condition will be used. Depending on what type of replicates the data are, users are recommended to either normalize the replicates or not before aggregating the replicates. For instance, if the replicates come from sequencing the same library multiple times, normalization might not be required before aggregating. However, if the replicates come from biological replicates, it is recommended to normalize between the replicates before pooling the replicates. Additional code in the future to handle replicates and consider variation between replicates might further make StripeDiff more useful. As the performance of StripeDiff is slightly decreased by noise signal, another potential way to improve the performance is to add algorithm for removing noise signal.

Although many Hi-C datasets have been generated in the last decade, the analysis of Hi-C data and functional interpretation of chromatin folding is still a challenge. Important features of chromatin folding are just starting to be observed in Hi-C data. These emerging features require novel bioinformatics methods and robust algorithms to investigate the underlying mechanisms. Chromatin stripe is a newly observed feature soon found to be functionally important and in a pressing need of robust tools for deep investigations. With the new toolkit StripeDiff, a user will be able to easily perform a genome-wide analysis to accurately define individual differential stripes. We further delivered the first tool for in silico generation of simulated Hi-C data embedded with differential chromatin stripes, and the first set of benchmarks to assess the performance of differential stripe detection. StripeDiff will act as a valuable tool to analyze differential stripes and facilitate the understanding of chromatin structure regulation in numerous biological processes and diseases.

## MATERIALS AND METHODS

### Stripe identification

We denote a matrix of chromatin contact frequency determined by Hi-C as *C* = {*c*_*i*, *j*_ : *i* = 1 : *n*, *j* = 1 : *n*}, where *c_i,j_* is the contact probability between bins *i* and *j* on a chromatin, and *n* is the total number of bins on the chromatin. As *C* is symmetric, we will describe the algorithm using the lower triangle of *C* unless otherwise specified (Fig. 1A, step a). We will also only describe the algorithm using a vertical stripe in the lower triangle as an example, as a horizontal stripe is simply the transpose of a vertical stripe. To detect the left and right edge of a vertical stripe, we created a matrix of edging signal as *E* = {*e*_*i*, *j*_ = *e*_*i* + 1, *j*_ − *e*_*i*, *j*_ : *i* = 1 : (*n* − 1), *j* = 1 : (*n* − 1)}. For a vertical window *k* that is *l* bins long in the column *i*, where *l* is a parameter provided by a user, the top and bottom boundaries of the window are *j = t* and *j = w = t + l* (Fig. 1A, step b). We calculated the mean edging signal of the window *k* as $m_{i,k}=\frac{1}{w-t}\sum_{\;j=t}^{w}e_{i,j}$ (Fig. 1A, step c). We next plotted *m*_*i*, *k*_ as a function of *i* and define the left and right edges of a vertical stripe *s* as an upward peak *U_s_* followed by a downward peak *D_s_* in the plot (Fig. 1A, step d). To detect the upward and downward peaks, we used the Loess regression to smoothen the vector of stripe edging signals. Thereafter, we defined a local maximum filter: For each location *i* on the stripe, the filter determines whether the value on location *i* is the maximum value when compared to all values of the *k* nearest locations on the left side and *k* nearest locations on the right side (*k* = 2). If the value of location *i* is the maximum value when compared to values of the *k* nearest locations on the left side and *k* nearest locations on the right side, we will define *i* as the location of an upward peak. Similarly, we defined a local minimal filter to detect downward peaks. We then further matched an upward peak to a downward peak located within *l* bins.

### Stripe length estimation

For a vertical stripe *s*, with the upward and downward peaks of the mean edging signal in a vertical window *k* being *U_s_* and *D_s_*, the location of the left and right edges are *i*_(*U_s_*)_ and *i*_(*D_s_*)_. The mean edging signals at the two edges in the vertical window *k* are *m*_*i*(*U_s_*), *k*_ and *m*_*i*(*D_s_*), *k*_. We define a stripe peak intensity signal for the stripe *s* in the window *k* as *P*_*s*, *k*_= ∣*m*_*i*( *U_s_*), *k*_ − μ_*i*( *U_s_*)_∣ + ∣*m*_*i*(*D_s_*), *k*_ − μ_*i*(*D_s_*)_∣, in which $\mu_{i}=\frac{1}{n}\sum_{\;j=1}^{n}e_{i,j}$ is the sample mean of stripe edging signal in the column *i* (Fig. 1A, step e). We next plotted *P*_*s*, *k*_ as a function of *k* and detected change points in the curve (Fig. 1A, step f) using the Segment Neighborhoods method (*31*). For a vertical stripe, the peak intensity should be stronger in the stripe when compared to the vertical region outside of the stripe. Therefore, the algorithm will detect a high peak intensity segment starting from the anchor location, and then followed by a low peak intensity segment. Because we used the Segment Neighborhoods method (*31*) to detect change point between the two segments (probability cutoff of 0.9), minor gaps will not be defined as change points. The stripe length will be the total length of all high-intensity segments detected at the anchor location.

### Differential stripe detection

The normalized values in a Hi-C contact matrix can be modeled as normally distributed Gaussian random variables (*31*, *32*). To have the distribution closer to a normal distribution (fig. S1), we further transformed the values to the log scale (Fig. 1A, step g). Therefore, the stripe edging signal *m*_*i*, *k*_ defined by us can also be modeled as following a log normal distribution *N*(μ, σ^2^), in which μ and σ are the mean and SD. We note that the parameters μ and σ are assumed to be consistent and independent of *i*. We assume that the upward and downward peaks of a stripe are both not differential between two samples, i.e., the null hypothesis. Evidence against the null hypothesis is taken as evidence for the difference between the two samples. In a window *k* that covers the full length of a stripe *s* in samples 1 and 2, *m*_*u*1_ = *m*_*i*( *U_s_*), *k*,1_ and *m*_*u*2_ = *m*_*i*( *U_s_*), *k*,2_ are height values of the upward peak, and *m*_*d*1_ = *m*_*i*(*D_s_*), *k*,1_ and *m*_*d*2_ = *m*_*i*(*D_s_*), *k*,2_ are those of the down peak.

Differential stripe was tested using H_0_: *m*_*u*1_ = *m*_*u*2_ and *m*_*d*1_ = *m*_*d*2_, and H_1_: *m*_*u*1_ ≠ *m*_*u*2_ or *m*_*d*1_ ≠ *m*_*d*2_. We evaluated these hypotheses by one-sided chi-square test based on $\hat{K}={\left[z_{u}\right]}^{2}+{\left[z_{d}\right]}^{2}$. Here, $z_{u}=\frac{m_{u}-\bar{m_{u}}}{\sigma_{u}}$, in which *m_u_* = log (*m*_*u*1_) − log (*m*_*u*2_), whereas $\bar{m_{u}}$ and σ*_u_* are the mean and SD of *m_u_*. Similarly, $z_{d}=\frac{m_{d}-\bar{m_{d}}}{\sigma_{d}}$, in which *m_d_* = log (*m*_*d*1_) − log (*m*_*d*2_), whereas $\bar{m_{d}}$ and σ*_d_* are the mean and SD of *m_d_*. Therefore, the variables that we were testing here are *z_u_* and *z_d_*. We used *z_u_* and *z_d_* instead of *m_u_* and *m_d_*, because *m_u_* and *m_d_* could follow different normal distributions. *z_u_* and *z_d_* represent *z* scores, which can be both modeled as standard normal distribution (mean value of 0 and SD value of 1).

We compared the performance between StripeDiff and edgeR, which were used to calculate the significance of differential chromatin interactions in each stripe between samples. We generated Hi-C simulation data for two conditions, each with two replicates. In edgeR analysis, we calculated the read count in each stripe region, which horizontally started from the left edge and ended at the right edge, and vertically started from the diagonal point and extended to the stipe terminal. Then, we used edgeR to calculate the false discovery rate of difference in read count from each stripe region between samples.

We used the overlapping ratio (Jaccard index) cutoff value of 0.5 between the predicted and simulated stripe anchors. The overlapping ratio was calculated as the intersection width divided by the union width between predicted and simulated stripe anchors. The predicted and simulated stripes are considered as overlapped if the overlapping ratio is larger than the cutoff.

### Simulation of Hi-C data

To simulate the count of chromatin contacts (fig. S1), we used the IMR90 Hi-C contact map as a reference. For each pairwise interaction (*i*, *j*), we first simulate the contact probability of each TAD as log(*M*_*i*, *j*_) = Norm(μor), which is a log normal distribution, and μ and σ are the mean and variation of contact probability in each TAD. Next, we select a few TADs to have a loop at the corner of each TAD. We further select TADs to have a stripe at the left or right boundary of each TAD. We also select TADs to each merge with the TAD next to them. These TADs were selected using IMR90 as a template to evaluate similarity to real data or randomly to evaluate performance of StripeDiff. In addition, μ and σ can be calculated from IMR90 data as the template or specified in the following way: μ ata[abs(*i* − *j*)] × slope + *m*, σ = {log [abs(*i* − *j*)] × slope_σ_ + *m*_σ_}^2^. Here, *i* and *j* are the smaller and larger genomic coordinates; *m*, slope, *m*_σ_, and slope_σ_ are the parameters to be specified. It is recommended to set slope as negative and slope_σ_ as positive values. The resolution is 10 kbp unless a different resolution was explicitly specified. The distribution of simulated stripe length mimics that in the IMR90 Hi-C data unless a length was explicitly specified. We did not simulate interchromosomal reads, so the simulated sequencing depth is the number of intrachromosomal reads.

### Hi-C data, ChIP-seq data, and RNA-seq data processing and analysis

All Hi-C data were processed and normalized by the software Juicer. Data were analyzed with a 10-kb resolution unless otherwise specified. ChIP-seq data were mapped to the hg19 reference genome for human cell lines and the mm9 reference genome for mouse cell lines by Bowtie version 1.1.0 (*51*), and peaks were called by DANPOS 2.2.3 with default parameters (*52*). Unless otherwise specified, the ChIP-seq signal of CTCF, Pol II, and H3K27me3 is the ChIP-seq read number calculated by DANPOS 2.2.3 at each base pair when shown in genome browser tracks or for each ChIP-seq enrichment peak when shown in boxplots. The number of ChIP-seq reads mapped to the whole genome was normalized by DANPOS2 to 25 million. In boxplot analysis of ChIP-seq signals at differential stripes, signals of all peaks located in the same stripe anchor were aggregated, and then the fold difference in the sum was calculated between samples, e.g., between the wild-type and CTCF mutant cell types. The reference genes UCSC Known Genes were downloaded from the UCSC Genome Browser website. The RNA-seq data were mapped to the hg19 reference genome using TopHat (version 2.1.1) with default parameters (*53*). The gene expression level and significance of differential expression were defined by Cuffdiff (version 2.0.12) based on the classical fragments per kilobase of exon per million mapped fragments (FPKM) values with default parameters (*54*).

To plot the average read density around a group of stripe anchors, we first categorized the stripes as vertical (left) stripes or horizontal (right) stripes based on the location in the contact map (Fig. 3D). For each stripe in the vertical stripe group, assuming that the stripe is *L* bins long, we retrieved a *W*-bin by *L*-bin region flanking the center of the stripe anchor, with *W*/2 bins by *L* bins at the left side and *W*/2 bins by *L* bins at the right side. If the vertical stripe group has *S* stripes, we will have a 3D matrix *M_T_* = [*c_wls_*], *w* = 1,2,3, …, *W*; *l* = 1,2,3, …, *L*; *s* = 1,2, …, *S*. Each value *c_wls_* in this matrix represents the number of read pairs at the dot (*w*, *l*) in the stripe *s*. We then converted the matrix *M_T_* to a 1D vector *V* = (*v_w_*), in which $v_{w}=\frac{1}{L\times S}\sum_{l=1}^{L}\sum_{s=1}^{S}c_{wls}$. A plot for the right group of strips will be generated in the same way after rotating the contact maps accordingly. The values in the vector were used to generate the read density plot for the left strip group. To plot the average read density heatmap for the left stripe group, we converted the 3D matrix *M_T_* to a 2D matrix *M_D_* = {*c_wl_*}, in which $c_{wl}=\frac{1}{S}\sum_{s=1}^{S}c_{wls}$. Another matrix was generated for the right stripe group in the same way after rotating the contact map accordingly. Thereafter, the two matrices were averaged to generate a single matrix, which was then used to plot the heatmap.

## Notes

View/request a protocol for this paper from *Bio-protocol*.
