# Supplementary material for: StripeDiff: Model-based algorithm for differential analysis of chromatin stripe
Source: Sci Adv. 2022 Dec 7;8(49):eabk2246. doi: 10.1126/sciadv.abk2246 (PMC9728969; doi:10.1126/sciadv.abk2246)
Supplement: Supplementary file 1 — Figs. S1 to S9 [file sciadv.abk2246_sm.pdf]

Science Advances
AAAS

Supplementary Materials for

StripeDiff: Model-based algorithm for differential analysis of chromatin stripe

Krishan Gupta *et al.*

Corresponding author: Kaifu Chen, kaifu.chen@childrens.harvard.edu

Sci. Adv. **8**, eabk2246 (2022)
DOI: 10.1126/sciadv.abk2246

This PDF file includes:

Figs. S1 to S9

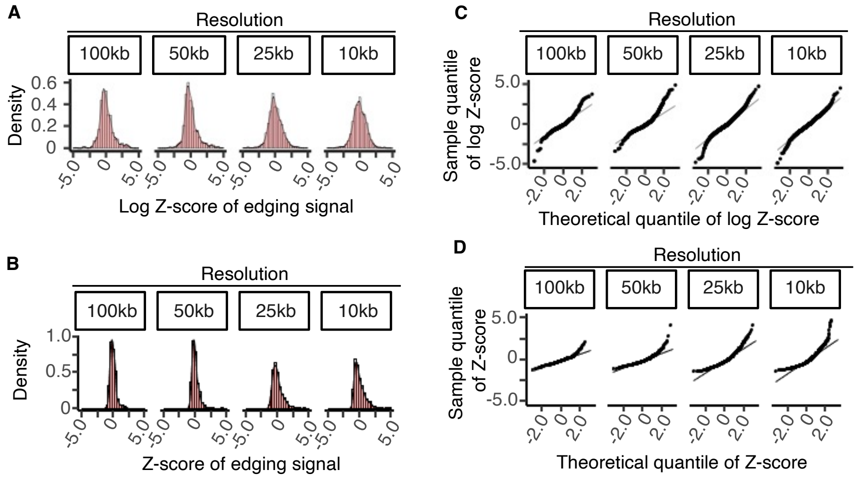

Fig. S1 Stripe edging signal can be modeled as a log normal distribution. (A-B). Probability density plot of log Z score (**A**) or Z score (**B**) of stripe edging signal. (**C-D**) Scatter plot to show correlation between observed log Z score value (**C**) or Z score value(**D**) and the values expected based on a normal distribution. Hi-C data from the IMR90 and HUVEC samples were combined for the analysis.

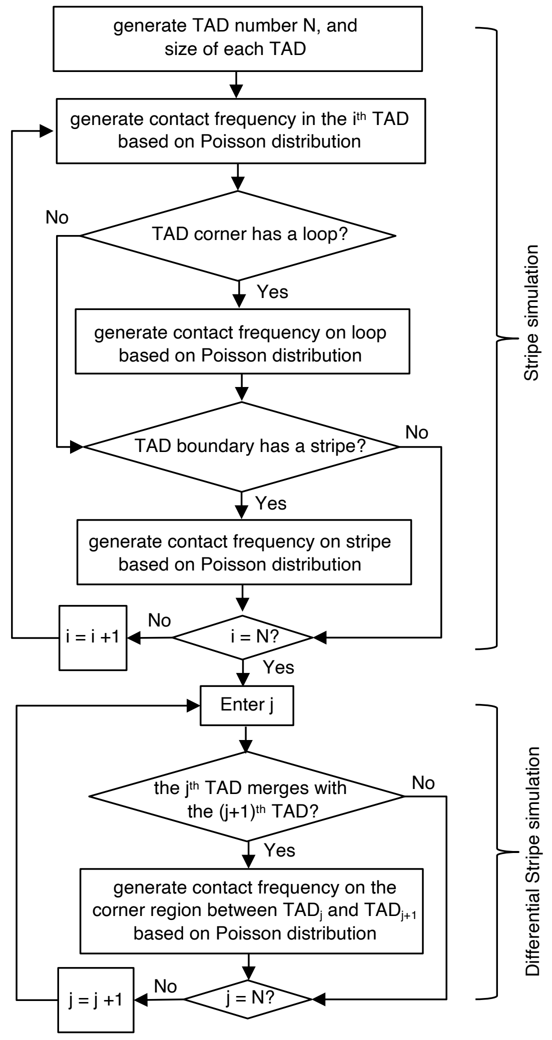

Fig. S2 A flowchart of the SimuStripe algorithm to simulate differential 3D genomes.

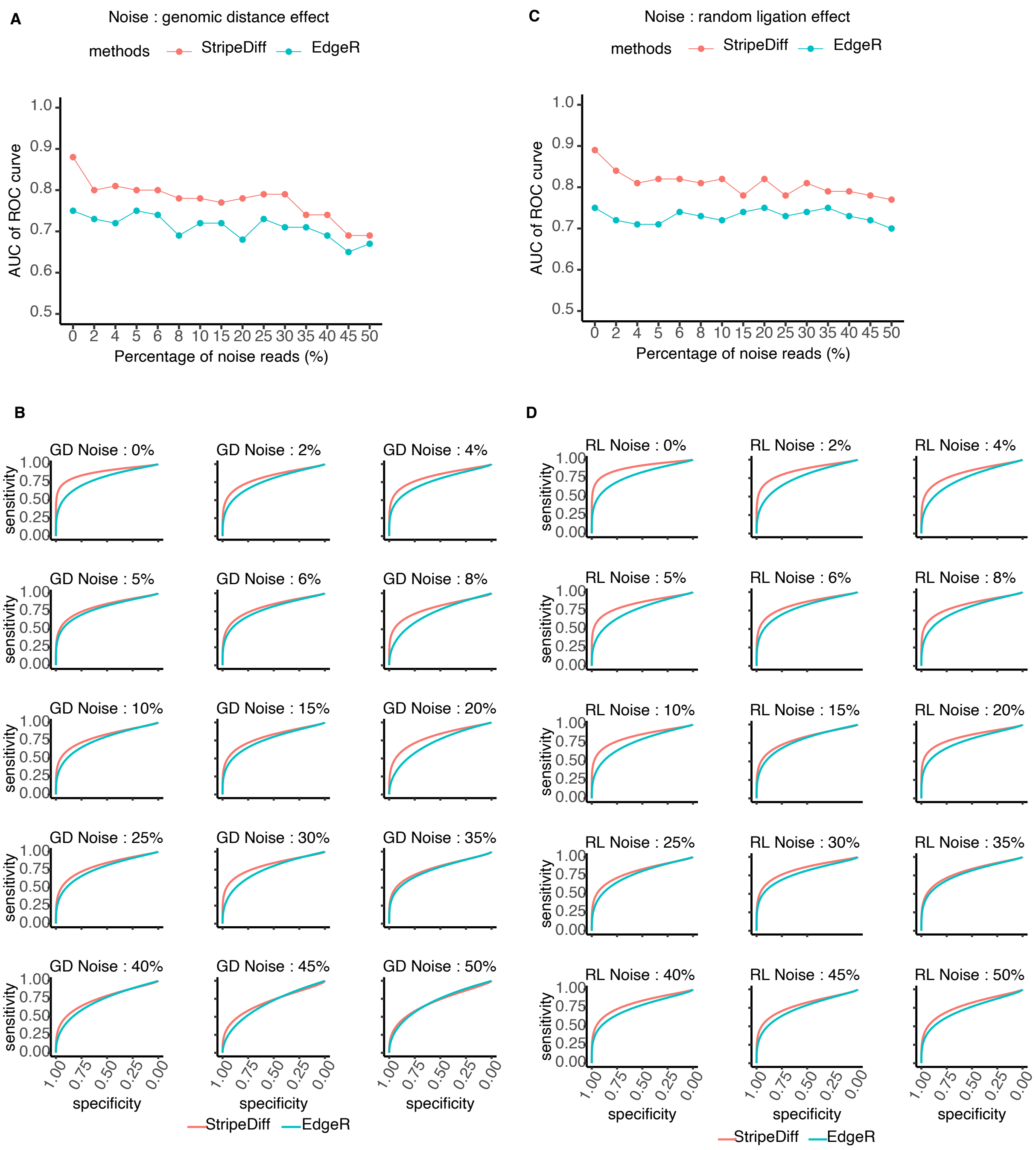

Fig. S3 StripeDiff is resistant to noise signal when applied to simulated data. (A, C) AUC of ROC curve plotted for datasets with different levels of noise signal. (**B, D**) ROC curves plotted for datasets with different levels of noise signal. Noise signal was simulated based on genomic distance (GD) effect (**A, B**) and random ligation (RL) effect in Hi-C protocol (**C, D**).

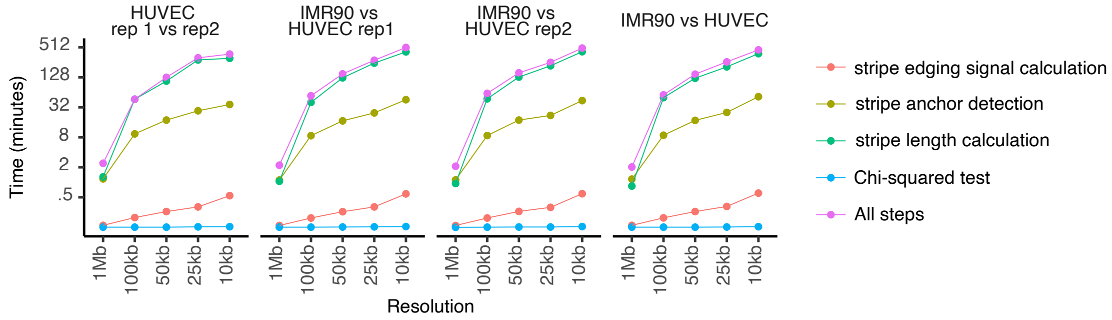

Fig. S4 The time that StripeDiff spent at individual steps of the algorithm when used to analyze real Hi-C data.

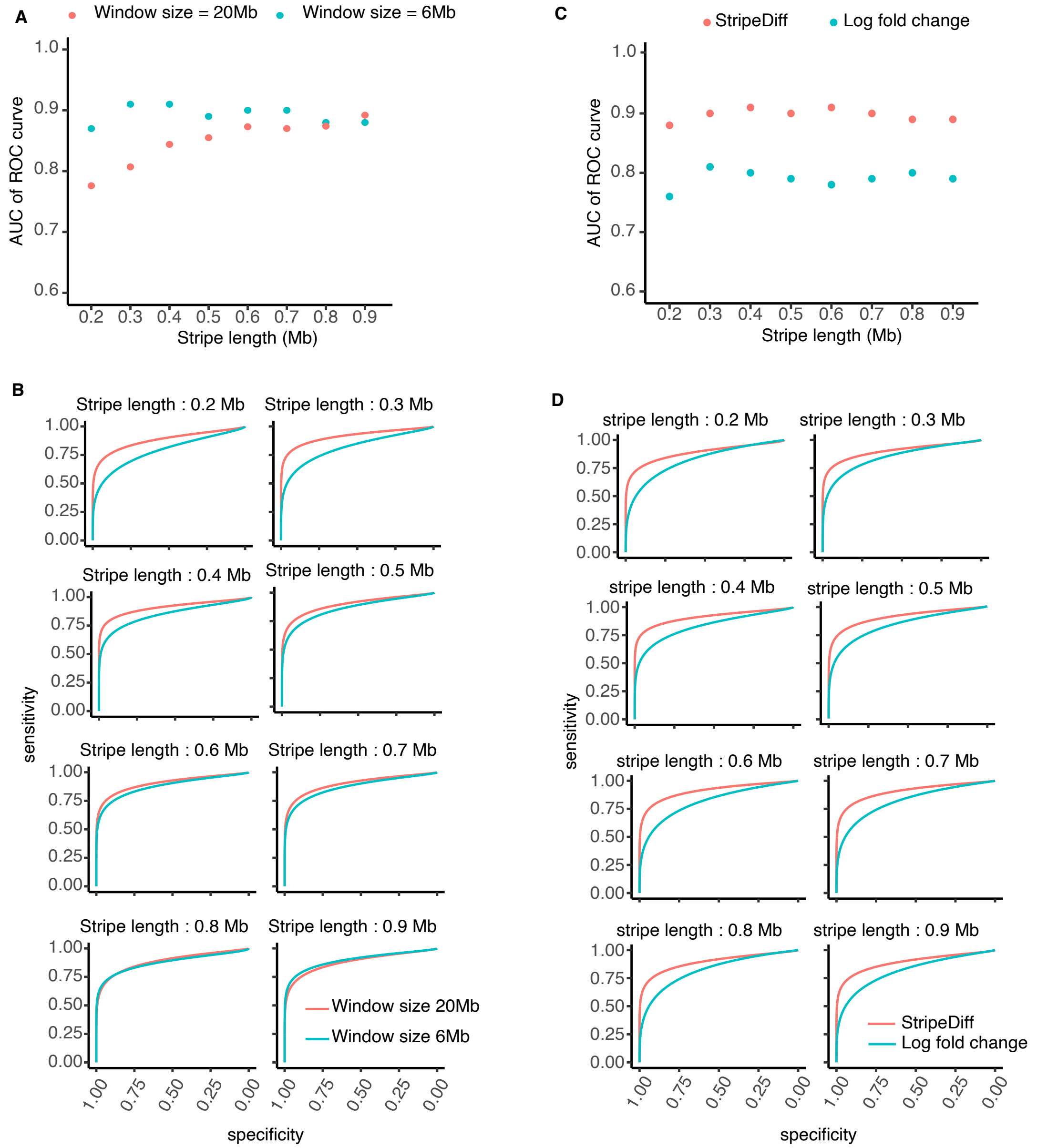

Fig. S5 StripeDiff showed robust performance when used to analyze simulated short stripes. (A, C) AUC of ROC curve plotted for stripes simulated to have different lengths. (**B, D**) ROC curves plotted for stripes simulated to have different lengths.

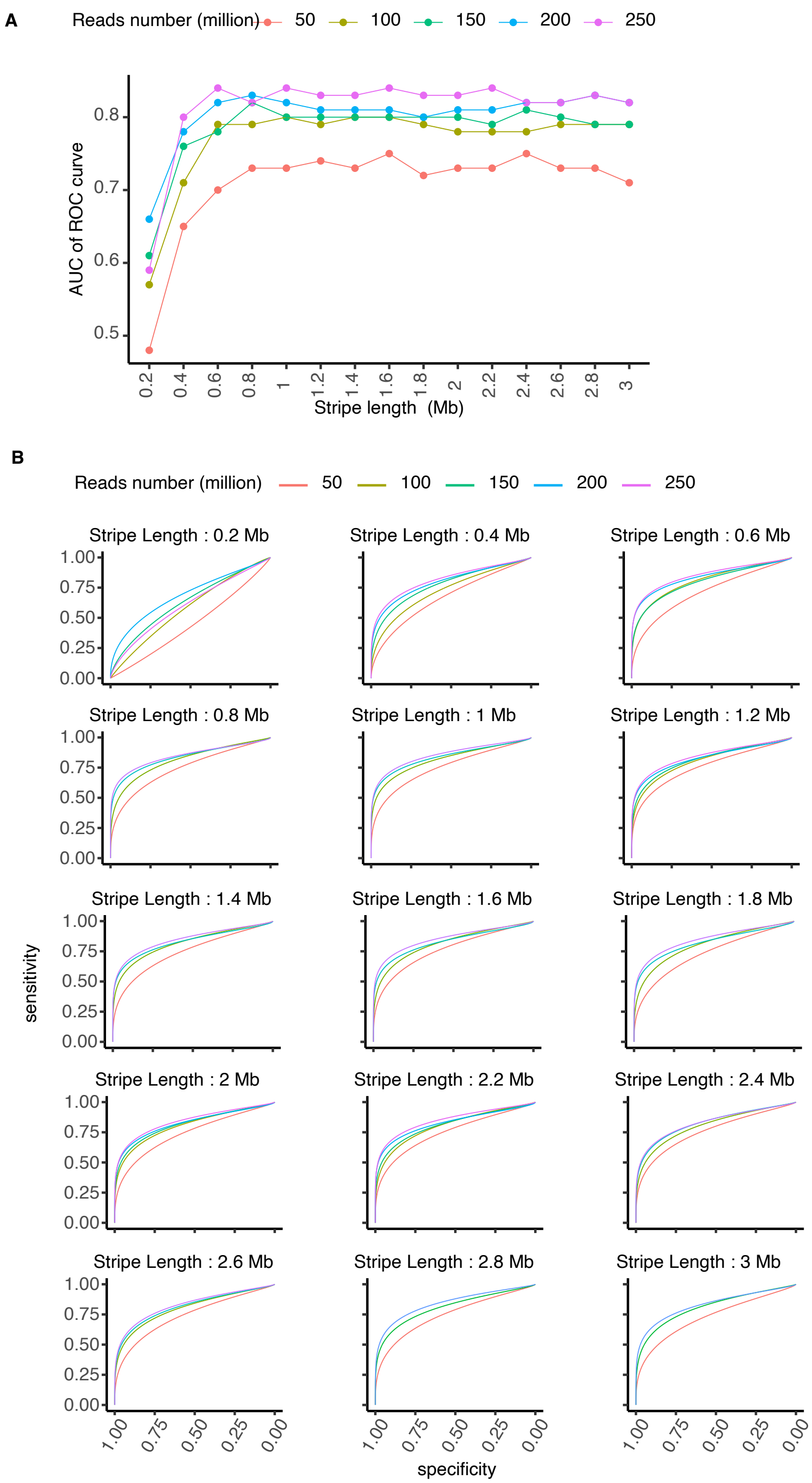

Fig. S6 StripeDiff showed robust performance when applied to simulated Hi-C datasets with different sequencing depth. (A) AUC of ROC curve plotted for datasets with different levels of noise signal and different sequencing depths. (**B, D**) ROC curves plotted for datasets with different levels of noise signal and different sequencing depths.

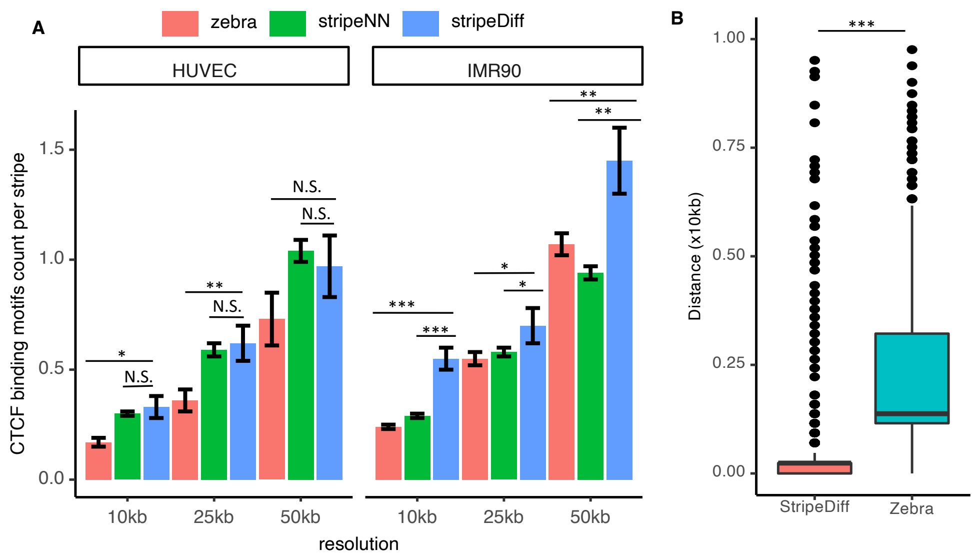

Fig. S7 StripeDiff show robust performance in identification of stripes in both simulated and real Hi-C data. (**A**) Bar plot to show number of CTCF motifs at anchor locations of identified stripes in the HUVEC and IMR90 dataset. (**B**) Boxplot to show distance between simulated stripe anchor locations and anchor locations of identified stripes. StripeNN was not tested for simulated data due to incompatible format of the simulated data. P value determined by one-tailed Wilcoxon rank test. *, $p < 0.05$; **, $p < 0.001$; ***, $p < 1e-10$.

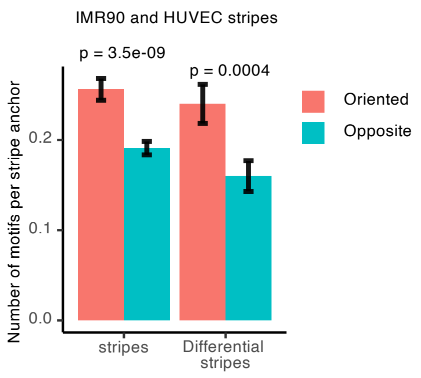

Fig. S8 Bar plot to show CTCF motif number at stripes or differential stripes identified by StripeDiff.

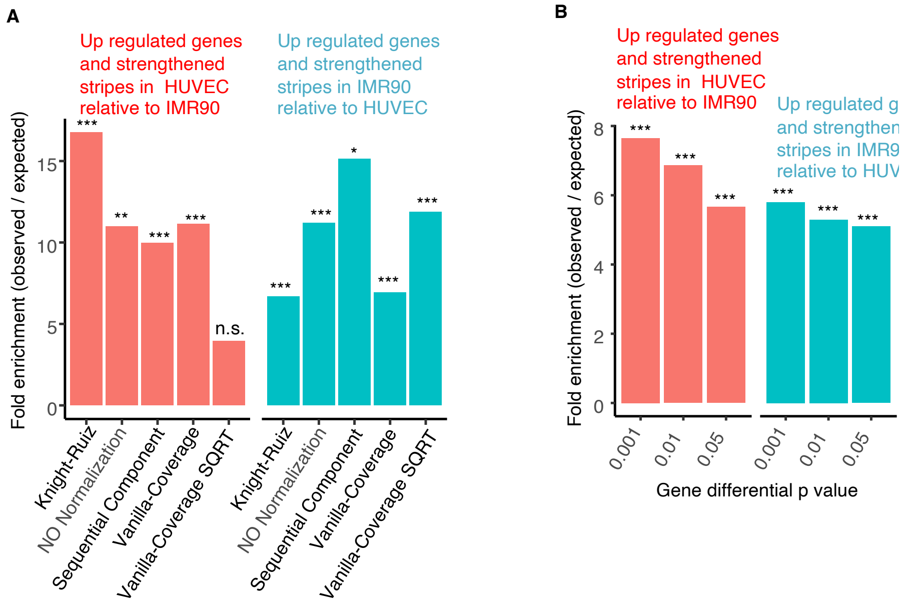

Fig. S9 Bar plot to show enrichment of up-regulated genes in strengthened stripes when different methods were used to normalize the Hi-C data (A) or different p value cutoffs were used to define differential genes (B). Fold enrichment is calculated as the observed gene number divided by gene number expected by random chance. Gene number expected by random chance is $N_r = N_s \times N_e / N$, in which N is the number of all genes in the genome, N_s and N_e are the number of genes associated with differential stripes and differential expression, respectively. P value of fold enrichment determined by Fisher's exact test. *, p < 1e-3; **, p < 1e-5; ***, p< 1e-10.
